# The *S. Cerevisiae* HAP Complex, a Key Regulator of Mitochondrial Function, Coordinates Nuclear and Mitochondrial Gene Expression

**DOI:** 10.1002/cfg.254

**Published:** 2003-02

**Authors:** S. Buschlen, J-M Amillet, B. Guiard, A. Fournier, C. Marcireau, M. Bolotin-Fukuhara

**Affiliations:** 1 Laboratoire de Génétique Moléculaire IGM Batiment 400. Université Paris Sud 91405 Orsay Cedex France; 2 Aventis Pharma Core Biotechnology/Yeast Genomics. 13 Quai Jules Guesde Vitry sur Seine 94403 France; 3 Centre de Génétique Moléculaire Avenue de la Terrasse Gif sur Yvette 91198 France

## Abstract

We have compared *Saccharomyces cerevisiae* global gene expression in wild-type and mutants (*Δhap2 and Δhap4*) of the HAP transcriptional complex, which has been shown to be necessary for growth on respiratory substrates. Several hundred ORFs are under positive or negative control of this complex and we analyse here in detail the effect of HAP on mitochondria. We found that most of the genes upregulated in the wild-type strain were involved in organelle functions, but practically none of the downregulated ones. Nuclear genes encoding the different subunits of the respiratory chain complexes figure in the genes more expressed in the wild-type than in the mutants, as expected, but in this group we also found key components of the mitochondrial translation apparatus. This control of mitochondrial translation may be one of the means of coordinating mitochondrial and nuclear gene expression in elaborating the respiratory chain. In addition, HAP controls the nuclear genes involved in several other mitochondrial processes (import, mitochondrial division) that define the metabolic state of the cell, but not mitochondrial DNA replication and transcription. In most cases, a putative CCAAT-binding site is present upstream of the ORF, while in others no such sites are present, suggesting the control to be indirect. The large number of genes regulated by the HAP complex, as well as the fact that HAP also regulates some putative transcriptional activators of unknown function, place this complex at a hierarchically high position in the global transcriptional regulation of the cell.


**Comparative and Functional Genomics**
*Comp Funct Genom* 2003; **4**: 37–46.
Published online in Wiley InterScience (www.interscience.wiley.com). **DOI:** 10.1002/cfg.254

**Conference Paper**

### The *S. cerevisiae* HAP complex, a key regulator of mitochondrial function, coordinates nuclear and mitochondrial gene expression

S. Buschlen[1], J-M Amillet[2], B. Guiard[3], A. Fournier[2], C. Marcireau[2] and M. Bolotin-Fukuhara[1]*

[1] *Laboratoire de Génétique Moléculaire, IGM, Batiment 400. Université Paris Sud, 91405 Orsay Cedex, France*
[2] *Aventis Pharma, Core Biotechnology/Yeast Genomics. 13 Quai Jules Guesde, 94403 Vitry sur Seine, France*
[3] *Centre de Génétique Moléculaire, Avenue de la Terrasse, 91198, Gif sur Yvette, France*

**Correspondence to:*
*M. Bolotin-Fukuhara, Laboratoire de Génétique Moléculaire, IGM, Batiment 400. Université Paris Sud, 91405 Orsay Cedex, France.*
*E-mail: bolotin@igmors.u-psud.fr*

Received: 26 August 2002
Accepted: 4 December 2002

### Abstract

**We have compared *Saccharomyces cerevisiae* global gene expression in wild-type and mutants (Δ*hap2* and Δ*hap4*) of the HAP transcriptional complex, which has been shown to be necessary for growth on respiratory substrates. Several hundred ORFs are under positive or negative control of this complex and we analyse here in detail the effect of HAP on mitochondria. We found that most of the genes upregulated in the wild-type strain were involved in organelle functions, but practically none of the downregulated ones. Nuclear genes encoding the different subunits of the respiratory chain complexes figure in the genes more expressed in the wild-type than in the mutants, as expected, but in this group we also found key components of the mitochondrial translation apparatus. This control of mitochondrial translation may be one of the means of coordinating mitochondrial and nuclear gene expression in elaborating the respiratory chain. In addition, HAP controls the nuclear genes involved in several other mitochondrial processes (import, mitochondrial division) that define the metabolic state of the cell, but not mitochondrial DNA replication and transcription. In most cases, a putative CCAAT-binding site is present upstream of the ORF, while in others no such sites are present, suggesting the control to be indirect. The large number of genes regulated by the HAP complex, as well as the fact that HAP also regulates some putative transcriptional activators of unknown function, place this complex at a hierarchically high position in the global transcriptional regulation of the cell. Copyright © 2003 John Wiley & Sons, Ltd.**

**Keywords:** ***Saccharomyces cerevisiae*; transcriptional regulation; *HAP2*; *HAP4*; mitochondrial function; respiration**

### Introduction

The yeast *Saccharomyces cerevisiae* has a predominantly fermentative metabolism. When grown on media containing glucose as carbon source, yeast cells repress their respiratory metabolism up to the point where all glucose has been consumed, leaving only ethanol as carbon source. In order to use ethanol, the cell has to reprogram its metabolism, a phase called 'diauxic shift'. Previous data have shown that the greater part of this reprogramming is under the control of the HAP complex.

The HAP complex is a heteromeric transcriptional regulator composed of four proteins. Hap2p, Hap3p and Hap5p associate to form the DNA-binding part, while Hap4p contains the activation domain (review in Forsburg and Guarente, 1989a). While *HAP2*, *HAP3*, *HAP5* are constitutively expressed, transcription of *HAP4* is induced during the diauxic shift (De Risi *et al.*, 1997). The

Copyright © 2003 John Wiley & Sons, Ltd.

HAP complex was originally identified as upregulating the expression of cytochrome *c* (Forsburg and Guarente, 1989a) and later on, of several genes encoding TCA cycle and respiratory chain enzymes (for review, see De Winde and Grivell, 1993).

During the global transcriptome analysis of yeast genes in relation to growth phase on glucose, De Risi *et al.* (1997) clustered genes according to their expression pattern and found that nuclear-encoded genes playing a role in respiratory chain function and TCA cycle were also transcriptionally induced at the diauxic shift, leading to the hypothesis that *HAP4* was responsible for this induction. The fact that *HAP4* plays a role in programming a respiratory metabolism is further confirmed by its metabolic effect when overexpressed (Blom *et al.*, 2000) and by its induction when yeast cells are placed in conditions of adaptative evolution under glucose-limiting conditions (Ferea *et al.*, 1999).

Despite these convergent observations, no precise identification of *HAP4* target genes has yet been made. We decided to address this question by comparing the transcriptome of three strains (a wild-type strain, a *hap2* deletion and a *hap4* deletion) during growth on galactose medium. Since the *hap2* and *hap4* deletion mutants are not able to grow on non-fermentable carbon sources, their effects on gene expression could only be examined on fermentable carbon sources. Galactose was chosen as carbon source to avoid glucose repression, which would have flattened out any possible effects. We report here the systematic analysis of genes related to mitochondrial function and biogenesis and show that the HAP complex positively controls the expression of genes involved in mitochondrial respiration and mitochondrial translation, thus allowing the coordinated expression of both genetic compartments.

### Materials and methods

### Strains and media

Complete deletions of the *HAP2* and *HAP4* genes were introduced into the W303-1A wild-type strain (Mat**a** ura3-1, trp1Δ2, leu2-3,112, his 3-11, ade2-1, can1-100), using the 'mass-murder' method developed by Fairhead *et al.* (1998) which employs kanMX4 (resistance to kanamycin in *Escherichia coli*, and to geneticin, 200 μg/l, in yeast) as selective marker (Wach *et al.*, 1994).

### RNA isolation

RNA extraction was done mechanically in liquid nitrogen as described by Hauser *et al.* (1998) from cultures grown in complete galactose medium [2.3% BactoAgar, 1% BactoPeptone, 1% Yeast Extract (Difco) and 2% Galactose]. Yeast cells (200 ml) grown to $OD_{600}$ 0.8–1.0 were pelleted. The pellet was then stirred a few seconds on a vortex and released as individual drops directly into liquid nitrogen, kept in the small Teflon vessel of a micro-dismembrator. After adding a 7 mm tungsten carbide bead, frozen drops were mechanically broken for 2 min at 3000 rpm. The frozen powder was instantly taken up in Trizol reagent (Invitrogen) and treated as instructed.

Three independent RNA preparations and labelling were made from three different cultures, for each of the three strains (WT, Δhap2 and Δhap4) in parallel.

### Probe generation and GeneChip hybridization

Experimental procedures for the GeneChip were performed as recommended by Affymetrix. Briefly, mRNA was isolated from total RNA with the Oligotex mRNA kit, as outlined by the manufacturer (Qiagen). Double-stranded cDNA was synthesized from mRNA with the SuperScript Choice system (Life Technologies) and a T7-(dT) 24 (GENSET) primer. *In vitro* transcription was performed on the cDNA to produce biotin-labelled cRNA with an Enzo Transcription Kit (Enzo). The biotinylated cRNA was cleaned with an RNeasy Mini Kit (Qiagen), fragmented to 50–200 nucleotide lengths, and then hybridized to Affymetrix YGS98 arrays. The arrays were then processed on the Affymetrix fluidics station and scanned on an HP GeneArray scanner.

### Data acquisition and analysis

Acquisition and quantification of array images were performed with Affymetrix Microarray Suite 4.0. RNA preparations from the wild-type and the deleted strains Δhap2 and Δhap4 were done three times, on different days and therefore from different cultures. All the data were normalized at the 75th percentile. The expression ratios were obtained using both normalized intensity and noise data through the PFOLD algorithm (Theilhaber *et al.*, 2002). Briefly, components of noise in the

Copyright © 2003 John Wiley & Sons, Ltd. 

intensities are identified and assumed to have normal distributions for both scans. An analytical expression is derived for the probability distribution function of intensities, using Bayes' theorem and the above assumptions about noise distribution. A joint probability distribution (JPD) of the fold change is derived using the above distributions. The JPD is used to calculate the median, confidence limits and $p$ values for the estimated fold-change. The fold change of a particular gene corresponds to the median of the fold changes deduced from the three biological replicates.

Raw data corresponding to this work can be found at: **http://mips.gsf.de/proj/medgen/mitop/**

### Results and discussion

### Transcriptome analysis reveals a large number of genes whose expression is dependent upon the presence of the HAP complex

The data point to the conclusion that the expression of several hundred genes is controlled directly or indirectly by the HAP complex. This large number of targets places this complex as an important and hierarchically highly-placed transactivator. About 230 genes are positively regulated (higher expression in the wild-type than in the Δ*hap2* and Δ*hap4* mutants), while about 240 seem to be negatively regulated. A closer examination, focused on respiratory metabolism, identifies the great majority of those genes upregulated in the wild-type as being directly involved in mitochondrial function and TCA cycle. Among the negatively regulated genes, the situation is quite different, since we found only two nuclear-encoded mitochondrial genes downregulated (negative effect of the Hap2p/Hap4p proteins), which are isoforms of positively-regulated ones. We will focus in this report only on those genes involved in mitochondrial function and biogenesis.

### The HAP complex controls the expression of genes coding for all the respiratory chain complexes and the enzymes of the TCA cycle

When De Risi *et al.* (1997) examined yeast global expression in relation to the growth phase, they observed around the diauxic shift an increase in expression of genes encoding enzymes of the TCA cycle and of the pathways that fuel substrates to it. This increase could not be directly attributed to *HAP4* as a general rule but this was strongly suspected, since *HAP4* itself was induced and since we and others have previously shown some of these genes to be regulated by the HAP complex (De Winde and Grivell, 1993; Dang *et al.*, 1994). In the experiments reported here, we found all the genes identified by De Risi *et al.* (1997) to be upregulated in the presence of the HAP complex (they showed two-fold higher expression in the wild-type than in the *hap2* and *hap4* mutants). These were *CIT2*, *CIT1*, *ACO1*, *MDH2*, *MLS1*, *ICL1*, *IDP2*, *MDH1*, *FUM1*, *SDH2*, *SDH3*, *SDH4*, *KGD1*, *KGD2*, *LPD1*, *IDH1*, as well as *PCK1*, *PYC1*, *PYC2*, *ALD2* and *ACS1* (Table 2, partA). It was expected that *IDH2* would be regulated, but we did not observe a significant effect of HAP. Another contradiction was the differential expression (ratio, $\Delta\text{hap2}:\text{wt} = 0.5$, where $\text{wt} = \text{wild-type}$) observed for *IDP1*, which was described as not carbon source-responsive by Haselbeck and McAlister-Henn (1993) and was not regulated in the De Risi *et al.* (1997) experiment. Except for these two cases, which have to be verified by other methods, it is now clear that the HAP complex controls the complete TCA cycle and related pathways. A coordinated increase in expression of about two- or three-fold in the wild-type as compared to the mutants was also observed for nuclear genes that code for the different components of the five respiratory chain complexes in yeast and some proteins (not all) involved in their assembly (Table 1). This co-response of different elements of the same functional families reinforces the interpretation that it reveals a mechanism allowing a good coordination of all transcripts when necessary. Related to this observation, two genes involved in heme biosynthesis, *HEM1* and *HEM2*, which had already been described as being controlled by *HAP2* (Keng *et al*, 1992; Keng and Guarente, 1987), were also found to be upregulated by a factor of 2.

Table 1 also shows that in statistically significant cases the regulatory effect is the same in both *hap2* and *hap4* mutants. In cases where isoenzymes exist, the HAP complex can exert different effects on their gene expression, probably in relation to the metabolic needs of the cell, e.g. the HAP genes were first isolated as a regulator of *CYC1* (iso1-cytochrome *c*), which is indeed upregulated in the wild-type strain in our transcriptome data. Conversely, *CYC7* (iso2-cytochrome *c*) is found

Copyright © 2003 John Wiley & Sons, Ltd. 

**Table 1.** Effect of the *HAP2* and *HAP4* deletion on the expression of nuclear genes encoding components and associated proteins of the five respiratory chain complexes of *S. cerevisiae*. In the column 'CCAAT site?'; 'yes' indicates the presence of a CCAAT sequence defined arbitrarily between −150 and −800 from the ATG and whatever the orientation. In the delta *hap2*/wt and delta *hap4*/wt columns, the ratio indicated is the median of the expression in the mutant divided by the median of the expression in the wild-type, these median coming from three independent experiments, made with different RNA preparations. SD, standard deviation. We considered data as significant only when $p < 0.001$. If this was not the case, the value is provided but is followed by an asterisk

| CCAAT site? | Gene name | Gene function | Δhap2/wt | SD | Δhap4/wt | SD |
|---|---|---|---|---|---|---|
| NADH dehydrogenase | | | | | | |
| Yes | *NDI1* | Mitochondrial NADH ubiquinone oxidoreductase | 0.23 | 0.03 | 0.38 | 0.02 |
| Yes | *CYB2* | Cytochrome *b*2 [L-lactate cytochrome c oxidoreductase] | 0.40 | 0.19 | 0.82 | 0.50 |
| Complex II | | | | | | |
| Yes | *SDH1* | Flavoprotein subunit of succinate dehydrogenase | 0.24 | 0.06 | 0.24 | 0.01 |
| Yes | *SDH2* | Succinate dehydrogenase (ubiquinone) iron-sulphur protein subunit | 0.29 | 0.04 | 0.33 | 0.11 |
| Yes | *SDH3* | Succinate dehydrogenase cytochrome *b* | 0.34 | 0.11 | 0.56 | 0.11 |
| Yes | *SDH4* | Succinate dehydrogenase membrane subunit | 0.39 | 0.04 | 0.41 | 0.10 |
| Yes | *TCM62* | Involved in assembly of complex II | 0.56* | 0.14 | 0.65* | 0.33 |
| Yes | *CYC1* | Iso-1-cytochrome *c* | 0.25 | 0.14 | 0.51 | 0.42 |
| Yes | *CYT1* | Cytochrome *c*1 | 0.41 | 0.03 | 0.47 | 0.02 |
| Complex III | | | | | | |
| Yes | *QCR1/COR1* | 44 kDa Core protein of yeast co-enzyme QH2 cytochrome *c* reductase | 0.26 | 0.01 | 0.39 | 0.02 |
| Yes | *QCR2* | 40 kDa Ubiquinol-cytochrome *c* reductase core protein 2 | 0.31 | 0.10 | 0.34 | 0.05 |
| Yes | *QCR6* | Ubiquinol-cytochrome *c* oxidoreductase subunit 6 (17 kDa) | 0.45 | 0.14 | 0.36 | 0.01 |
| Yes | *QCR7* | Ubiquinol-cytochrome *c* oxidoreductase subunit 7 (14 kDa) | 0.57 | 0.11 | 0.57 | 0.12 |
| Yes | *QCR8* | Ubiquinol-cytochrome *c* reductase subunit 8 (11 kDa protein) | 0.63 | 0.06 | 0.68 | 0.01 |
| Yes | *QCR9* | 7.3 kDa Subunit 9 of the ubiquinol cytochrome *c* oxidoreductase complex | 0.56 | 0.08 | 0.57 | 0.15 |
| Yes | *QCR10* | 8.5 kDa Subunit of the ubiquinol-cytochrome *c* oxidoreductase complex | 0.21 | 0.06 | 0.29 | 0.08 |
| Yes | *RIP1* | Rieske iron–sulphur protein of the mitochondrial cytochrome *bc*1 complex | 0.40 | 0.13 | 0.47 | 0.16 |
| Complex IV | | | | | | |
| Yes | *COX10* | Putative farnesyl transferase required for heme A synthesis | 0.49 | 0.18 | 1.17* | 0.41 |
| Yes | *COX11* | Putative heme A biosynthetic enzyme involved in forming the formyl group | 0.66 | 0.15 | 0.91* | 0.09 |
| Yes | *COX12* | Subunit VIb of cytochrome *c* oxidase | 0.38 | 0.15 | 0.48 | 0.02 |
| Yes | *COX13* | Subunit VIa of cytochrome *c* oxidase; may specifically interact with ATP | 0.47 | 0.14 | 0.51 | 0.14 |
| Yes | *COX14* | Mitochondrial membrane protein | 0.86 | 0.20 | 1.23 | 0.20 |
| Yes | *COX15* | Cytochrome oxidase assembly factor | 0.87 | 0.41 | 1.10 | 0.32 |
| Yes | *COX17* | Mitochondrial copper shuttle required for cytochrome *c* oxidase biogenesis | 0.79 | 0.08 | 0.80 | 0.24 |
| Yes | *COX18* | Involved in assembly of cytochrome *c* oxidase | 0.92* | 0.04 | 1.16 | 0.09 |

Copyright © 2003 John Wiley & Sons, Ltd. 

**Table 1.** Continued

| CCAAT site? | Gene name | Gene function | Δhap2/wt | SD | Δhap4/wt | SD |
|---|---|---|---|---|---|---|
| Yes | *COX4* | Subunit IV of cytochrome *c* oxidase | 0.27 | 0.07 | 0.34 | 0.11 |
| Yes | *COX5A* | Cytochrome *c* oxidase chain Va | 0.42 | 0.11 | 0.54 | 0.20 |
| Yes | *COX5B* | Cytochrome *c* oxidase chain Vb | 1.55 | 0.49 | NS | 0.15 |
| Yes | *COX6* | Subunit VI of cytochrome *c* oxidase | 0.31 | 0.08 | 0.36 | 0.08 |
| Yes | *COX7* | Subunit VII of cytochrome *c* oxidase | 0.52 | 0.23 | 0.62 | 0.09 |
| Yes | *COX8* | Cytochrome *c* oxidase chain VIII | 0.32 | 0.06 | 0.57 | 0.11 |
| Yes | *COX9* | Subunit VIIa of cytochrome *c* oxidase | 0.32 | 0.09 | 0.42 | 0.14 |
| Yes | *COX20* | Involved in assembly of cytochrome *c* oxidase | 0.51 | 0.08 | 0.51 | 0.07 |
| Yes | *MBA1* | Involved in assembly of cytochrome *c* oxidase | 0.46 | 0.09 | 0.62 | 0.10 |
| Complex V | | | | | | |
| Yes | *ATP1* | Mitochondrial F1F0-ATPase $\alpha$ subunit | 0.51 | 0.07 | 0.67 | 0.09 |
| Yes | *ATP2* | Mitochondrial F1F0-ATPase $\beta$ subunit | 0.45 | 0.14 | 0.45 | 0.08 |
| Yes | *ATP3* | Mitochondrial F1F0-ATPase $\gamma$ subunit | 0.35 | 0.06 | 0.46 | 0.10 |
| Yes | *ATP4* | Mitochondrial F1F0-ATPase $\Delta$ subunit | 0.50 | 0.08 | 0.45 | 0.10 |
| Yes | *ATP5* | Mitochondrial F1F0-ATPase subunit 5 | 0.35 | 0.09 | 0.36 | 0.10 |
| Yes | *ATP7* | Mitochondrial F1F0-ATPase $\Delta$ subunit | 0.27 | 0.03 | 0.31 | 0.07 |
| No | *ATP10* | Essential for assembly of a functional mitochondrial ATPase complex | 0.8* | 0.14 | 0.7* | 0.21 |
| Yes | *ATP11* | Essential for assembly of a functional F1-ATPase | 0.95 | 0.09 | 1.11 | 0.01 |
| Yes | *ATP12* | Essential for assembly of a functional F1-ATPase | 0.54 | 0.13 | 0.75 | 0.27 |
| Yes | *ATP13/AEP2* | Required for expression of F0 subunit 9 | 0.48* | 0.25 | 1.05* | 0.56 |
| Yes | *ATP14* | Mitochondrial F1F0-ATPase subunit h | 0.27 | 0.13 | 0.36 | 0.10 |
| Yes | *ATP15* | Mitochondrial F1F0-ATPase $\epsilon$ subunit | 0.46 | 0.08 | 0.63 | 0.05 |
| Yes | *ATP16* | Mitochondrial F1F0-ATPase subunit | 0.36 | 0.13 | 0.34 | 0.09 |
| Yes | *ATP17* | Mitochondrial F1F0-ATPase subunit f | 0.28 | 0.11 | 0.43 | 0.05 |
| Yes | *ATP20* | Mitochondrial F1F0-ATPase subunit g | 0.22 | 0.09 | 0.36 | 0.07 |
| Yes | *ATP21*/TIM11 | Mitochondrial F1F0-ATPase subunit e | 0.49 | 0.03 | 0.52 | 0.05 |
| Yes | *ATP18?* | Protein associated to the mitochondrial F1F0-ATPase | 0.43 | 0.05 | 0.69 | 0.07 |

to be downregulated (2.3-fold less expression in the wild-type than in the mutants), as would be expected for a gene which is expressed in anaerobiosis. *MDH1* and *MDH2*, encoding respectively the cytoplasmic and mitochondrial forms of malate dehydrogenases, are both regulated by HAP (ratio of 0.4 and 0.5, respectively) while *MDH3*, the peroxisomal isoform, does not seem to be regulated (0.8), an observation perhaps reflecting the need to co-regulate the two elements of the redox–malate shuttle. Transcriptome experiments do not tell us whether the regulatory effect is direct (binding of the transactivator upstream of the gene) or indirect (through a cascade). A 'CCAAT' binding site (arbitrarily chosen between −150 and −800 from the translation start) is present upstream of practically all the genes described in this table, but this does not necessary mean that this site is functional.

### Mitochondrial gene expression also seems to vary in function of the HAP complex

The Affymetrix yeast DNA chips also contain oligomers from mitochondrially-encoded elements of the respiratory chain. We found some of them to be also upregulated by a factor of 2–3. Two facts argue against a direct control of HAP on the mitochondrial genes. Transactivators associated to *pol*II are located in the nucleus and can be translocated into the cytoplasm but there is no evidence so far for mitochondrial localization. Only one transcription factor, *XTC1*, has a dual localization, but its mitochondrial function is different (Traven *et al.*, 2002). Furthermore, direct mitochondrial transcriptional regulation has not been observed in yeast, while information pointing to translational control does exist, particularly

Copyright © 2003 John Wiley & Sons, Ltd.

**Table 2.** List of all other genes regulated by *HAP2*. The experiments and the criteria are the same as those defined in Table 1. Only data with the Δhap2/wt ratio are provided, since no statistical differences were observed in Δhap4/wt as compared to Δhap2/wt. SD, standard deviation. We considered the data as significant only when $p < 0.001$. If this was not the case, the value is provided but is followed by an asterisk

| Gene | Function | Δhap2 : wt | SD |
|---|---|---|---|
| Part A: TCA cycle and related pathways | | | |
| *IDP2* | Cytosolic form of NADP-dependent isocitrate dehydrogenase | 0.27 | 0.05 |
| *PYK2* | Pyruvate kinase; glucose-repressed isoform | 0.32 | 0.18 |
| *PCK1* | Phosphoenolpyruvate carboxylkinase | 0.34 | 0.08 |
| *KGD2* | Component of α ketoglutarate dehydrogenase | 0.34 | 0.06 |
| *ACO1* | Mitochondrial aconitase | 0.35 | 0.06 |
| *FUM1* | Mitochondrial and cytoplasmic fumarase | 0.39 | 0.10 |
| *ICL1* | Isocitrate lyase | 0.39 | 0.09 |
| *KGD1* | α-Ketoglutarate dehydrogenase | 0.41 | 0.06 |
| *MLS1* | Carbon-catabolite sensitive malate synthase | 0.41* | 0.18 |
| *MDH1* | Mitochondrial malate dehydrogenase | 0.44 | 0.10 |
| *LPD1* | Dihydrolipoamide dehydrogenase precursor | 0.48 | 0.11 |
| *CIT1* | Citrate synthase. Nuclear encoded mitochondrial protein | 0.49 | 0.06 |
| *PYC1* | Pyruvate carboxylase | 0.49 | 0.17 |
| *IDP1* | Mitochondrial form of NADP-specific isocitrate dehydrogenase | 0.51 | 0.06 |
| *ACS1* | Inducible acetyl-coenzyme A synthetase | 0.51 | 0.15 |
| *ALD2* | Aldehyde dehydrogenase; (NAD(P)+) | 0.53 | 0.17 |
| *MDH2* | Cytosolic malate dehydrogenase | 0.54 | 0.09 |
| *IDH1* | Subunit of mitochondrial isocitrate dehydrogenase | 0.58 | 0.08 |
| *CIT2* | Non-mitochondrial citrate synthase | 0.65 | 0.05 |
| *ICL2* | Isocitrate lyase | 0.71* | 0.31 |
| *MDH3* | Malate dehydrogenase | 0.79 | 0.06 |
| *IDH2* | NAD+-dependent isocitrate dehydrogenase | 0.83 | 0.01 |
| Part B: mitochondrial translation apparatus | | | |
| *MEF2* | Mitochondrial elongation factor G-like protein | 0.25 | 0.08 |
| *RPM2* | Subunit of mitochondrial RNase P | 0.26 | 0.08 |
| *TUF1* | MtTranslation elongation factor Tu | 0.45 | 0.06 |
| *MSD1* | MtAspartyl-tRNA synthetase | 0.46 | 0.26 |
| *MRPL6* | Mt ribosomal protein MRPL6 | 0.51 | 0.12 |
| *MRPL19* | Mt ribosomal protein of the large subunit | 0.54 | 0.16 |
| *MSR1* | Arginyl-tRNA synthetase | 0.54 | 0.27 |
| *YMR31* | Mitochondrial ribosomal protein | 0.54 | 0.19 |
| *MRPS5* | Probable mitochondrial ribosomal protein S5 | 0.55 | 0.21 |
| *MRP51* | Mt ribosomal protein (small subunit) | 0.56 | 0.21 |
| *MRPL7* | Mt ribosomal protein MRPL7 | 0.56 | 0.06 |
| *YML15* | Mt ribosomal protein MRPL15 | 0.56 | 0.17 |
| *RML2* | Mt ribosomal protein L2 | 0.57 | 0.14 |
| *MEF1* | Mt elongation factor G-like protein | 0.58 | 0.14 |
| *ISM1* | Mt isoleucyl-tRNA synthetase | 0.58* | 0.32 |
| *PET123* | Mt ribosomal protein of small subunit | 0.59 | 0.25 |
| *MRF1* | Mt polypeptide chain release factor | 0.59 | 0.15 |
| *MRPL11* | Mt ribosomal protein MRPL11 | 0.59 | 0.10 |
| *MST1* | Mt threonine-tRNA synthetase | 0.6 | 0.22 |
| *MSE1* | Mt glutamyl-tRNA synthetase | 0.6 | 0.14 |
| *SLS1* | Coupling of mitochondrial translation and transcription | 0.60* | 0.11 |
| *YMR188C* | Weak similarity to bacterial ribosomal protein S17 | 0.61 | 0.14 |
| *YPR081C* | Strong similarity to glycyl-tRNA synthetases | 0.61 | 0.38 |
| *MSM1* | Mt methionyl-tRNA synthetase | 0.62 | 0.18 |
| *NAM9* | Putative mt S4 ribosomal protein | 0.62 | 0.22 |
| *YER087W* | Similarity to *E. coli* prolyl-tRNA synthetase | 0.62 | 0.30 |
| *MRP4* | Mt ribosomal protein homologous to E. coli S2 | 0.64 | 0.07 |
| *MRPL40* | Mt ribosomal protein MRPL40 | 0.64 | 0.11 |
| *MRPL9* | Mt ribosomal protein MRPL9 | 0.64 | 0.06 |
| *MRPL35* | Mt ribosomal protein MRPL35 | 0.64 | 0.03 |

Copyright © 2003 John Wiley & Sons, Ltd. 

**Table 2.** Continued

| Gene | Function | Δhap2 : wt | SD |
|---|---|---|---|
| Part B: mitochondrial translation apparatus (Continued) | | | |
| *MRPL10* | Mt ribosomal protein MRPL10 | 0.65 | 0.02 |
| *MRPL17* | Ribosomal protein of the large subunit (YmL30) | 0.66 | 0.08 |
| *NAM2* | Mt leucyl tRNA synthetase | 0.66 | 0.37 |
| *YMR158W* | Weak similarity to *E. coli* ribosomal S8 protein | 0.66 | 0.33 |
| Part C: mitochondrial dynamics | | | |
| *DNM1* | Dynamin-related protein | 0.46 | 0.10 |
| *YOR227W* | Similarity to microtubule-interacting protein Mhp1p | 0.58 | 0.41 |
| *MMM1* | Mitochondrial structure and inheritance | 0.58 | 0.02 |
| Part D: mitochondrial protein folding and import | | | |
| *MIR1* | Mitochondrial import receptor | 0.41 | 0.07 |
| *MAS2* | 53 kDa Subunit of the Mt processing protease | 0.51* | 0.09 |
| *TIM44* | 48.8 kDa protein involved in Mt protein import | 0.52 | 0.05 |
| *XDJ1* | Homolog of E. coli DnaJ; closely related to *UDJ1* | 0.53 | 0.30 |
| *MDJ1* | Involved in mt biogenesis and protein folding | 0.54 | 0.16 |
| *AFG1* | Related to YTA10-12 | 0.55 | 0.04 |
| *YTA12* | Assembly and degradation of mitochondrial membrane complexes | 0.55 | 0.12 |
| *PIM1* | Mitochondrial ATP-dependent protease | 0.58 | 0.16 |
| *SSC1* | Mitochondrial matrix protein involved in protein import | 0.64 | 0.17 |
| *MSF1′* | Probably involved in intramitochondrial protein sorting | 0.60* | 0.33 |
| *SSE2* | MtHSP70 family member | 0.65 | 0.91 |
| *TOM70* | Mt specialized import receptor of the outer membrane | 0.65 | 0.16 |
| Part E: regulatory proteins | | | |
| *YBR033W* | Probable regulatory Zn-finger protein | 0.32 | 0.09 |
| *YJL103C* | Putative regulatory protein | 0.34 | 0.07 |
| *YKR075C* | Weak similarity to negative regulator Reg1p | 0.61 | 0.17 |
| *YPR013C* | Similarity to transcription factors | 2.07 | 1.21 |
| *YBR267W* | Probable Zn-finger protein (C2H2 type) | 2.42 | 0.35 |
| *YER130C* | Similarity to Msn2p and weak similarity to Msn4p | 2.46 | 1.01 |
| Part F: mitochondrial carriers/translocators | | | |
| *ODC1* | Mitochondrial oxodicarboxylate carrier protein | 0.28 | 0.06 |
| *YFR045W* | Similarity to mitochondrial citrate transport proteins | 0.55 | 0.06 |
| *PET9/AAC2* | Mitochondrial ADP/ATP translocator | 0.65 | 0.07 |
| *AAC3* | Mitochondrial ADP/ATP translocator | 2.04* | 0.81 |
| Part G: miscellaneous | | | |
| *SNZ2* | Pyridoxin biosynthesis? Resistance to oxygen singlet toxicity? | 0.12 | 0.12 |
| *OM45* | 45 kDa Mitochondrial outer membrane protein | 0.25 | 0.10 |
| *YDL085W* | Strong similarity to NADH dehydrogenase | 0.31 | 0.52 |
| *SOD2* | Mt Manganese-containing superoxide dismutase | 0.41 | 0.06 |
| *YLR164W* | Strong similarity to Sdh4p C | 0.46* | 0.19 |
| *MSS51* | Protein involved in processing and translation of COX1 | 0.54 | 0.24 |
| *MBR1* | MBR1 protein precursor, function unknown | 0.62 | 0.11 |
| *MSS116* | Promotes ATP-dependent splicing of yeast group II intron | 0.66 | 0.12 |

through translational activators that seem to be limiting (Green-Willms *et al.*, 2001), and a coupling between mRNA synthesis and/or processing and mRNA maturation via the *SLS1* gene (Bryan *et al.*, 2001). If a (indirect) regulatory effect of HAP exists on mitochondrial gene expression, the simplest explanation would be that HAP partly controls the expression of chromosomal genes involved in mitochondrial translation. The observed increase in mRNAs would reflect their possible stabilization (e.g. as proposed above via the *SLS1* gene). A closer examination does indeed favour this hypothesis, since the key translation factors (genes encoding initiation factor *TUF1*, elongation factors *MEF1* and *MEF2*, termination factor *MRF1*) are all upregulated by a factor of 2–4. The *SLS1*

Copyright © 2003 John Wiley & Sons, Ltd. 

gene, probably involved in the coupling between transcription and translation, could also be regulated, but the *p* value was not robust enough to conclude this with confidence. Several other genes controlling tRNA maturation (*RPM2*, upregulated four times) or mitochondrial aminoacyl tRNA synthetases are in the same situation. We examined genes encoding mitochondrial ribosomal proteins and found some of them to be also upregulated by a factor of 2 (*MRPL6*, *MRPL19*, *MRPS5*, *MRPL7*, *PET123*, *MRPL11*, *YMR188c* and *NAM9*), but it remains to be understood why other ribosomal genes are not regulated similarly.

### Other mitochondrial genes under control of the HAP complex

Table 2 provides the list of all *S. cerevisiae* ORFs found to be regulated by HAP in these studies and involved in mitochondrial biogenesis and function. All these genes are regulated by a factor of 2–3, and controlled in the same way by the Hap2p (Table 2) and the Hap4p (data not shown) proteins. Apart from genes coding for elements of the mitochondrial translation apparatus (see above), other groups of genes are also informative on the role of this transactivator. The HAP complex controls elements of the mitochondrial import pathway, focusing essentially on chaperones (probably reacting to the increased amount of nuclear proteins that have to be imported for respiratory functions) and especially the TIM44/SSE2 complex, regarded as a central motor unit of the mitochondrial import machinery (reviewed in Rassow *et al.*, 1995). A few genes related to mitochondrial shape and movement but not to mitochondrial inheritance in general are also under the control of HAP. This result is consistent with the differences observed for mitochondrial shape in relation to glucose repression (discussed in Church and Poynton, 1998). Finally, in addition to some genes for which functions are not clearly defined, or which are homologous to components of the respiratory chain, a few mitochondrial carriers (for citrate, oxodicarboxylate and ATP/ADP) are also regulated. The case of ATP/ADP carriers again reflects the differential expression of the three isoforms, as previously described. It was shown that *AAC2*, the main carrier, was regulated by HAP (Betina *et al.*, 1995); *AAC1* was regulated by heme and oxygen (Gavurnikova *et al*, 1996); and *AAC3* was regulated by carbon source but not by the HAP complex (Sokolikova *et al.*, 2000). In our work, we indeed found that *AAC1* was not regulated, while *AAC3* was downregulated by *HAP2*. This last effect is certainly indirect, since Sokolikova *et al.* (2000) have shown that the responsive sequence of the repressor is not the HAP binding site and may be mediated by other transactivators.

### The expression of some putative transactivators of unknown function is regulated by HAP

With respect to the last point discussed in the previous paragraph, it is interesting to note that several putative transactivators were found to be controlled by *HAP2*, some of them possessing a degenerate 'CCAAT box'. This effect remains to be confirmed, especially since for some of them the standard deviation is quite large. Transcriptome analysis of these functionally uncharacterized transactivators in simple (Δtransactivator) or double (ΔtransactivatorΔhap2) mutants should help to understand their exact place in these regulatory cascades.

### What is the function of HAP4 in evolution?

*HAP4* mRNA is strongly induced at the diauxic shift, practically undetectable in glucose and expressed in glycerol and galactose. As already shown by others (Forsburg and Guarente, 1989b) and confirmed by our data, Hap4p is the moiety required for reprogramming of the cell from fermentation to respiration. Because this role is linked to the characteristic metabolism of *S. cerevisiae*, and because *HAP4* orthologues had not yet been identified in other species, it could even be hypothesized that *HAP4* would be unique to this yeast. More recently, we isolated *KlHAP4*, the *HAP4* gene of the yeast *Kluyveromyces lactis* (Bourgarel *et al.*, 1999), a yeast which has a respiratory metabolism and which is poorly glucose-repressed (Ferrero *et al.*, 1978; Breunig, 1989). The other components of the complex (*KlHAP2*, *KlHAP3*, *KLHAP5*) have also been characterized (Nguyen *et al.*, 1994; Mulder *et al.*, 1994; Bourgarel, 2000). The question of the function of the HAP complex in *K. lactis* (and possibly in other yeasts) is therefore opened. In *K. lactis*, the nuclear genes involved in mitochondrial function (such as *KlCYC1*, *KlQCR7* or *KlQCR8*) are constitutively expressed (Freire-Picos *et al.*, 1995) and, up to now, no genes, with

Copyright © 2003 John Wiley & Sons, Ltd. 

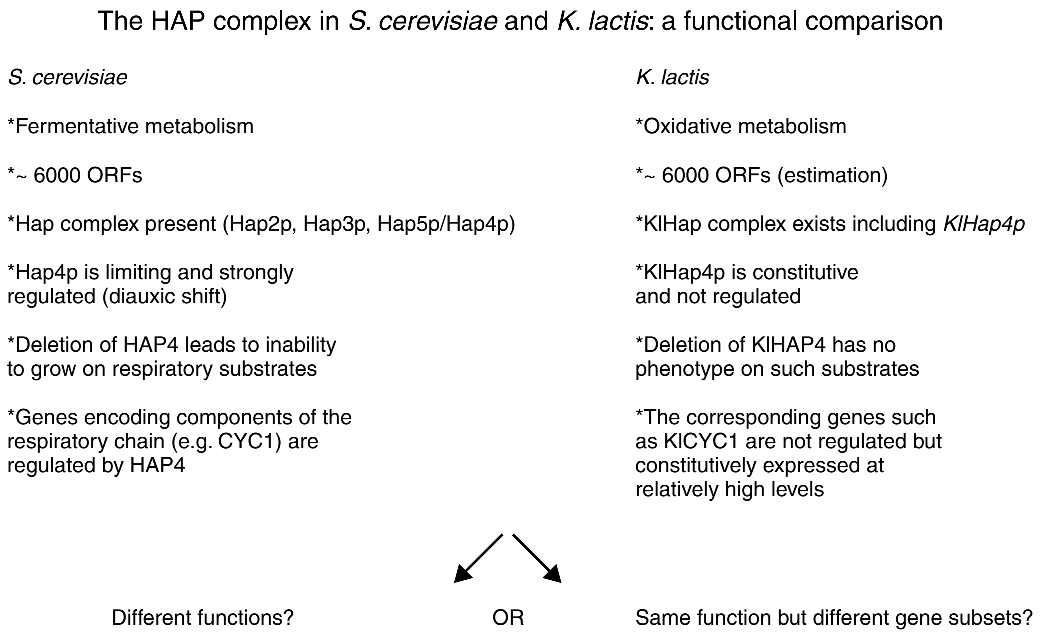

**Figure 1.** Schematic representation of the functional differences between the HAP complexes of the two yeasts *Saccharomyces cerevisiae* and *Kluyveromyces lactis*

the exception of the D-lactate ferricytochrome *c* reductase (Lodi *et al*, 1998) have been shown to be controlled by the complex (see also Mulder *et al*, 1995). Furthermore, the disruption of each of these *K. lactis* genes (or even of the double mutant *Klhap4 Klhap2*, data not shown) has not resulted in any respiratory-deficient phenotype. A summary of the functional differences between these two yeasts is presented in Figure 1. The recent developments of the Genolevures project (2000), which will provide enough gene sequences to test large-scale regulation, is now opening the way to a comparative approach of the functional evolution of regulatory networks.

### Acknowledgements

This work has been supported by the European Commission (Contract 'GARNISH' No. QLK3-2000-00174 and 'MitEURO' No. QLG1-CT-2001-00966).

### References

Betina S, Gavurnikova G, Haviernik P, Sabova L, Kolarov J. 1995. Expression of the *AAC2* gene encoding the major mitochondrial ADP/ATP carrier in *Saccharomyces cerevisiae* is controlled at the transcriptional level by oxygen, heme and HAP2 factor. *Eur J Biochem* **229**: 651–657.

Blom J, Teixera de Mattos IM, Grivell LA. 2000. Redirection of the respiro-fermentative flux distribution in *Saccharomyces cerevisiae* by overexpression of the transcription factor Hap4p. *Appl Environ Microbiol* **66**: 1970–1973.

Bourgarel D, Nguyen CC, Bolotin-Fukuhara M. 1999. *HAP4*, the glucose-repressed regulated subunit of the HAP transcriptional complex involved in the fermentation-respiration shift, has a functional homologue in the respiratory yeast *Kluyveromyces lactis*. *Mol Microbiol* **31**: 1205–1215.

Bourgarel D. 2000. Etude comparative du facteur de transcription multimérique Hap2/3/4/5p chez les levures *Saccharomyces cerevisiae* et *Kluyveromyces lactis*: caractérisation des domaines fonctionnels de la sous-unité régulatrice ScHap4p. Thèse de l'Institut National Agronomique Paris-Grignon.

Breunig KD. 1989. Glucose repression of LAC gene expression in yeast is mediated by the transcriptional activator *LAC9*. *Mol Gen Genet* **216**: 422–427.

Bryan AC, Rodeheffer MS, Wearn CM, Shadel GS. 2002. Sls1p is a membrane-bound regulator of transcription-coupled processes involved in *Saccharomyces cerevisiae* mitochondrial gene expression. *Genetics* **160**: 75–82.

Church C, Poyton RO. 1998. Neither respiration nor cytochrome *c* oxidase affects mitochondrial morphology in *Saccharomyces cerevisiae*. *J Exp Biol* **201**: 1729–1737.

De Winde JH, Grivell LA. 1993. Global regulation of mitochondrial biogenesis in *Saccharomyces cerevisiae*. *Progr Nucleic Acids Res* **46**: 51–91.

Dang VD, Valens M, Bolotin-Fukuhara M, Daignan-Fornier B. 1994. A genetic screen to isolate genes regulated by the yeast CCAAT-box binding protein Hap2p. *Yeast* **10**: 1273–1283.

De Risi JL, Iyer VR, Brown P. 1997. Exploring the metabolic and genetic control of gene expression on a genomic scale. *Science* **278**: 680–686.

Copyright © 2003 John Wiley & Sons, Ltd. 

Fairhead C, Thierry A, Denis F, Eck M, Dujon B. 1998. 'Mass-murder' of ORFs from three regions of chromosome XI from *Saccharomyces cerevisiae*. *Gene* **223**: 33–46.

Génolevures project. 2000. *FEBS Lett* **487**(1): special issue.

Ferea T, Botstein D, Brown PO, Rozenzweig RF. 1999. Systematic changes in gene expression patterns following adaptive evolution in yeast. *Proc Natl Acad Sci USA* **96**: 9721–9726.

Freire-Picos MA, Hollenberg CP, Breunig KD, Cerdan ME. 1995. Regulation of cytochrome *c* expression in the aerobic respiratory yeast *Kluyveromyces lactis*. *FEBS Lett* **360**: 39–42.

Ferrero I, Rossi C, Landini MP, Puglisi PP. 1978. Role of the mitochondrial protein synthesis in the catabolite repression of the petite negative yeast *Kluyveromyces lactis*. *Biochem Biophys Res Commun* **80**: 340–348.

Forsburg SL, Guarente L. 1989a. Communication between mitochondria and the nucleus in regulation of cytochrome genes in the yeast *Saccharomyces cerevisiae*. *Ann Rev Cell Biol* **5**: 153–180.

Forsburg SL, Guarente L. 1989b. Identification and characterization of *HAP4*: a third component of the CCAAT-bound *HAP2/HAP3* heteromer. *Genes Dev* **3**: 1166–1178.

Gavurnikova G, Sabova L, Kissova I, Haviernik P, Kolarov J. 1996. Transcription of the *AAC1* gene encoding an isoform of mitochondrial ADP/ATP carrier in *Saccharomyces cerevisiae* is regulated by oxygen in a heme-independent manner. *Eur J Biochem* **239**: 759–763.

Green-Willms NS, Butler CA, Dunstan HM, Fox TD. 2001. Pet111p, an inner membrane-bound translational activator that limits expression of the *Saccharomyces cerevisiae* mitochondrial COX2. *J Biol Chem* **276**: 6392–6397.

Haselbeck RJ, McAlister-Henn L. 1993. Function and expression of yeast mitochondrial NAD- and NADP-specific isocitrate dehydrogenases. *J Biol Chem* **268**: 12116–12122.

Hauser NC, Vingron M, Scheideler M, *et al*. 1998. Transcriptional profiling on all open reading frames of *Saccharomyces cerevisiae*. *Yeast* **14**: 1209–1221.

Keng T, Richard C, Larocque R. 1992. Structure and regulation of yeast *HEM3*, the gene for porphobilinogen deaminase. *Mol Gen Genet* **234**: 233–243.

Keng T, Guarente L. 1987. Constitutive expression of the yeast *HEM1* gene is actually a composite of activation and repression. *Proc Natl Acad Sci USA* **84**: 9113–9117.

Liu Z, Butow RA. 1999. A transcriptional switch in the expression of yeast tricarboxylic acid cycle genes in response to a reduction or loss of respiratory function. *Mol Cell Biol* **19**: 6720–6728.

Lodi T, Goffrini P, Bolondi I, Ferrero I. 1998. Transcriptional regulation of the KlDLD gene, encoding the mitochondrial enzyme D-lactate ferricytochrome *c* oxidoreductase in *Kluyveromyces lactis*: effect of Klhap2 and fog mutations. *Curr Genet* **34**: 12–20.

Mulder W, Scholten IH, de Boer RW, Grivell LA. 1994. Sequence of the *HAP3* transcription factor of *Kluyveromyces lactis* predicts the presence of a novel 4-cysteine zinc-finger motif. *Mol Gen Genet* **245**: 96–106.

Mulder W, Scholten I, Grivell L. 1995. Distinct transcriptional regulation of a gene coding for a mitochondrial protein in the yeasts *Saccharomyces cerevisiae* and *Kluyveromyces lactis* despite similar promoter structures. *Mol Microbiol* **17**: 813–824.

Nguyen C, Bolotin-Fukuhara M, Wesolowski-Louvel M, Fukuhara H. 1994. The respiratory system of *Kluyveromyces lactis* escapes from *HAP2* control. *Gene* **152**: 113–115.

Rassow J, Voos W, Pfanner N. 1995. Partner proteins determine multiple functions of Hsp70. *Trends Cell Biol* **5**: 207–212.

Sokolikova B, Sabova L, Kissova I, Kolarov J. 2000. A carbon-source-responsive element is required for regulation of the hypoxic ADP/ATP carrier (*AAC3*) isoform in *Saccharomyces cerevisiae*. *Biochem J* **352**: 893–898.

Theilhaber J, Bushnell S, Jackson A, Fuchs R. 2001. Bayesian estimation of fold-changes in the analysis of gene expression: the PFOLD algorithm. *J Comput Biol* **8**: 585–614.

Traven A, Staresincic L, Arneric M, Sopta M. 2002. The yeast protein Xtc1p functions as a direct transcriptional repressor. *Nucleic Acids Res* **30**: 2358–2364.

Wach A, Brachat A, Pohlmann R, Philippsen P. 1994. New heterologous modules for classical or PCR-based gene disruptions in *Saccharomyces cerevisiae*. *Yeast* **10**: 1793–1808.

Copyright © 2003 John Wiley & Sons, Ltd. 
